# Collectivism and meaning-making: A search for moderators

**DOI:** 10.1371/journal.pone.0346979

**Published:** 2026-04-30

**Authors:** Daphna Oyserman, Yahan Huang, Amabel Y. Jeon

**Affiliations:** University of Southern California, Los Angeles, California, United States of America; Seton Hall University, UNITED STATES OF AMERICA

## Abstract

People differ in how much they endorse collectivistic values (e.g., valuing group membership, and experiencing as essential in-group belonging, adhering to norms, and group connection). A posited behavioral consequence of valuing collectivism is that people may attempt to avoid group ruptures by actively seeking meaning in what their interaction partners say, asking themselves, “How might this make sense?” when a statement is ambiguous. Indeed, people who endorse collectivism find more meaning in ambiguous claims made by others. We investigate the robustness of this association and examine three theory-central potential moderators (communicator group membership, focus on meaning-making vs assessing accuracy, processing depth; *N* = 1,174). Across three experiments and in pooled analyses, higher collectivism is associated with rating ambiguous statements as more meaningful; this relationship is stronger when the communicator is from an in-group rather than an out-group, supporting the first posited moderator. We do not find support for the second moderator, perhaps due to the subtlety of our meaning vs. accuracy manipulation. And, while higher later incidental recall of communicator group membership is associated with finding more meaning in ambiguous statements, this incidental processing main effect is not consistently moderated by collectivism. Exploratory pooled analyses also suggest that people drew more meaning from ambiguous statements from a communicator who was a fellow student at their alma mater or at the rival university, rather than from a more contentious social group (their own or the rival political party). Moreover, at least just before and after a Presidential election in which Democrats were underdogs, collectivistic Republicans saw more meaning in messages from both Republicans and Democrats. Collectivistic Democrats saw more meaning only in messages from other Democrats. Our findings suggest that collectivism’s effects on meaning-making are context-dependent --group type and group boundary salience shape effect sizes. Future research should consider this interplay.

## Collectivism and meaning-making: A search for moderators

Humans need other humans to survive; evolutionary and cultural psychologists assume that human culture and evolution co-evolved to address recurring problems arising from needing to band together [1–3]. Banding together requires knowing who is in the group and a means of signaling group membership (collectivism). The implication is that connection is fundamental to well-being and manifests as a motivation to build and maintain relationships through engaging with others [4–6]. Indeed, being able to infer meaning and intent, to “read between the lines” and understand what is not explicitly stated is a valued trait (e.g., “social intelligence” [7,8], “reading the air” [9] in Japanese *kuuki-wo-yomu*, and “eye-measure” [10,11] Korean *nunchi*). Collectivism is associated with high-context communication in which making sense of the message requires that the listener actively process what was said and what was implied by nonverbal, contextual, and other features [12,13]. People who value collectivism may actively seek meaning by searching for confirmatory evidence (e.g., asking themselves: “How might what this person said make sense?”) [14]. While not yet tested, collectivists may be more likely to find meaning if the speaker’s group membership is salient, given the association between collectivism and the salience of category-based group memberships [15]. Across three studies, we both replicate prior research documenting that collectivism is associated with finding meaning in ambiguous statements and advance this research by testing underlying processing using three key moderators proposed in the literature (group membership, meaning making, depth of processing). Before describing our methods and results, we provide an overview of the relevant literature.

### Culture, collectivism, and communication

Culture can be studied at three levels of analysis: as a set of human universals, including evolved signals of group membership [16], as a set of chronic between-society, region, or country differences in the salience of a particular aspect of this universal human culture (e.g., varying levels of collectivistic values or patterns of behavior, [4]), or as between-person variability in endorsement of relevant values (e.g., collectivistic values, [17–20]). At each of these levels of analysis, collectivism highlights the centrality of groups [21,22], group boundaries [23], and group membership [24]. People invest in their groups; in-group favoritism is a universal aspect of culture [25,26]. As might be expected, it is more salient in societies and people who endorse collectivistic values and practices [27,28]. Collectivism is associated with sensitivity to in-group-related information [29] and to social context, and with centralizing relationships [30–33]. Collectivists are also more attentive to contextual and relational elements in communication [34–36]. When collectivism is on the mind, people focus more on relationships with their group members; they are more attuned to social context and to what others may be trying to communicate [37–39].

Moreover, perhaps because collectivism fosters focus on staying within the group, it is associated with indirect communication. To make sense of the speaker’s intent in this form of communication, message recipients must maintain a high level of context-awareness [40]. Empirically, collectivists tend to find meaningful patterns in what others might view as random variation [41,42]. This is the case whether collectivism is inferred from country-level indicators of collectivism, measured at the individual level as endorsement of collectivistic values, or manipulated such that people momentarily infer that they endorse collectivistic values [14].

The broader literature suggests three key theory-based moderators of the association between collectivism and finding meaning in ambiguous communications. First, collectivism is associated with the salience of group membership. All things being equal, listeners assume speakers are cooperative communicators, sharing information they believe is true, unbiased, and useful to the listener [43,44]. People who endorse collectivism may infer that a message comes from an in-group member attempting to provide them with relevant information. Or they may be particularly attuned to making sense of what potentially threatening out-groups are trying to communicate. The implication is that if group membership is provided, it should moderate the collectivism-to-meaning-making relationship. Of course, people are members of multiple groups, and some are likely to be more central or self-relevant than others. Groups that are not central, or even irrelevant, to the self may be less likely to moderate the association between collectivism and meaning-making [45,46].

A second possible moderator is an active search for meaning. Prior research documents an association between collectivism and context-dependent communication (the kind of communication that requires listeners read between the lines to infer meaning) as well as with finding meaning in ambiguous claims [14]. This latter finding is robust across different ways of operationalizing collectivism: as an individual difference measure, a between-country comparison, or a momentary experience [14]. In the latter case, after researchers led people to believe that they endorsed collectivistic values more, they subsequently rated ambiguous statements as more meaningful, yielding some evidence of causality [14]. Moreover, when asked to describe what statements mean, people higher in collectivism who rated them as more meaningful also provide a more meaningful interpretation of the statement [14]. Yet, we did not find an empirical test of the assumption that collectivism triggers an active search for meaning (vs assessing whether a statement is accurate). We address this here. If collectivism is associated with an automatic search for meaning, then making this mental procedure accessible should moderate the effect of collectivism on meaning-making. If collectivism triggers a search for meaning, the unique association between collectivism and finding meaning should be attenuated if this mental procedure (rather than a plausible alternative, processing for truth value) is triggered in context.

A related, third possible moderator entails depth of processing. Memory, operationalized as recall of information content and source, depends, in part, on attentional resources applied during encoding [47,48], which itself is a function of current goals, assumptions, and values [49,50]. In the case of collectivism, these goals may include processing for both communicator group membership and for the content communicated. On average, people preferentially process in-group-related stimuli [51–54] and have better memory for in-group-related events and information [55–58]. When asked to describe what the communicator intends when shown an ambiguous statement, higher collectivists actively construct possibly meaningful content while lower collectivists report that the statement is empty of content [14]. Though, to our knowledge, this has not been directly tested, group membership should be a particularly strong processing trigger for people higher in collectivism. Specifically, the relationship between collectivism and finding meaning in ambiguous statements should, in part, be a function of deeper processing as revealed in later, better incidental recall of what was said (message content) and by whom (group membership of message communicator). Because group membership matters, people higher in collectivism should also be more likely to retain this information.

### Groups that matter and information processing

Confirmatory processing for meaning is a double-edged sword; it can help people “read between the lines,” but it can also lead people to find meaning even in random information [59]. Evidence suggests that while people’s cognitive biases are not shaped by their group membership, group membership affects which messages people attend to and may affect the relative weight they place on source credibility vs. message content in evaluating messages. Relevant to the current studies, for example, within political psychology, similar biases are displayed by Democrats and Republicans when they evaluate evidence germane to their political positions [60]. Members of both political parties engage in identity-protective motivated cognition [61], are susceptible to ideologically congruent misinformation [62,63], and to forming false memories that reflect their partisan narratives [64]. At the same time, Republicans and Democrats differ in their selective exposure [65] and the importance they place on source credibility versus message content when assessing political communications [66]. Hence, we explore whether each of our moderators functions in the same way among Democrats and Republicans.

### Current studies and protocol

Our group membership markers were university affiliation (Study 1, University of Southern California undergraduate students [following [67]]) and political identity (Studies 2–3, American voters on Prolific following [60,68]). In Study 1, we included one group (University of Southern California undergraduates); in Studies 2 and 3 we included two groups (Republicans and Democrats), allowing us to test follow-up exploratory questions by pooling across studies.

#### Research questions.

RQ1: Is collectivism associated with finding meaning in ambiguous claims?
RQ2: Does group membership information moderate the association between collectivism and meaning-making?
RQ3: Does mental procedure moderate the association between collectivism and meaning-making? If collectivism triggers seeking meaning, can we see this via a diminished association with collectivism among those instructed to apply this mental procedure rather than an alternative one (seek accuracy)?
RQ4: Does depth of processing moderate the association between collectivism and meaning-making? If collectivism triggers deeper processing, is the association stronger among those who did so as operationalized by better content and source recall?
EQ: Are the patterns observed in RQ1-RQ4 consistent for Democrats and Republicans?

**Fig 1:**
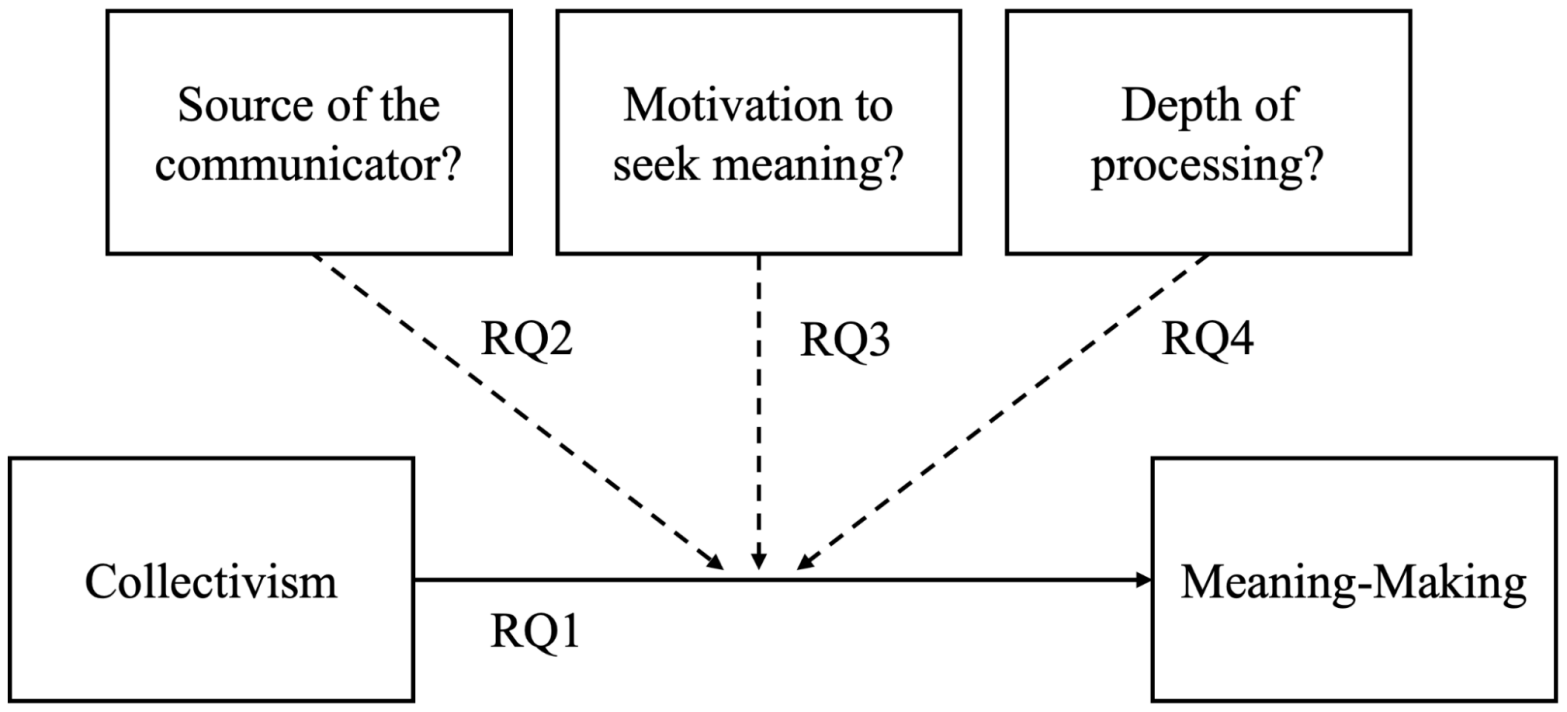
Conceptual Model: Collectivism-to-Meaning-Making Relationship (Solid Line) with Potential Moderators as Dashed Lines.

Fig 1 graphically represents our conceptual model of the relationship between collectivism and finding meaning (RQ1) and potential moderators. If the relationship is a function of group membership, then speaker source should affect how meaningful people find their communications (RQ2). If it is due to the search for meaning, instructions to find meaning should attenuate this relationship (RQ3). And, if it is due to deeper processing (RQ4), the relationship should be stronger among those with better source or content recall.

#### Participants.

The pre-test, pilot, and Study 1 were conducted with undergraduates in the university subject pool who participated for credit. Studies 2 and 3 were conducted with Prolific adults who had voted in the 2020 (Study 2) or the 2024 (Study 3) U.S. Presidential elections for the Republican or the Democratic candidate. Participants read the consent form on Qualtrics; only those who clicked their consent could continue to the study. All procedures were approved by the University of Southern California Institutional Review Board (#UP-20–00982-AM001, American Voters Participants for study #UP-20–00982, Does this statement apply to my life? IRB Study 1 #UP-20–00982; Studies 2 and 3 #UP-20–00982-AM001). We report the total collected sample size (*N*) and the final analyzed sample after exclusions (*n*). Pre-test: *N* = 99; 63.6% female; Age *M* = 19.94, *SD* = 1.18; see S1 Table); Pilot: *N* = 212, *n* = 205; 62.0% female; Age *M* = 20.19, *SD* = 2.10; see S1 Table); Study 1: *N* = 420, *n* = 405, 64.0% Female; Age *M* = 20.36, *SD* = 2.81; see S2 Table); Study 2: Trump voters (*N* = 198, *n* = 193; 48.1% female; Age *M* = 43.87, *SD* = 14.03) and Biden voters (*N* = 200, *n* = 192; 58.9% female; Age *M* = 40.64, *SD* = 12.69; see S3 Table). Study 3: Trump voters (*N* = 200, *n* = 194; 50.0% female; Age *M* = 38.75, *SD* = 12.63) and Harris voters (*N* = 200, *n* = 190; 67.9% female; Age *M* = 38.66, *SD* = 12.96; see S3 Table). For each study, we excluded participants who did not complete the full survey and those whose response completion time exceeded the outer fence of completion time: Q3 + 3 x IQR of completion time, as these extreme time outlier participants likely did not complete the survey in one sitting. This resulted in 7 exclusions from the Pilot, 15 from Study 1, 13 from Study 2 (5 Trump voters, 8 Biden voters), and 16 from Study 3 (6 Trump voters, 10 Harris voters). For simplicity, we will refer to Trump supporters as Republicans and Biden/Harris supporters as Democrats.

#### Transparency.

Pre-test data collection began on February 11, 2022, and ended on April 4, 2022 (Pilot November 3, 2022 to December 12, 2022. Study 1, January 18, 202,3 to March 14, 2023, data collection for Studies 2 (October 31, 2024) and Study 3 (March 11, 2025) completed in a single day. We used Qualtrics software and conducted all surveys online. We pre-registered our Study 2 predictions and analyses at https://aspredicted.org/5fbm-5sns.pdf. All stimuli, data, syntax, additional online materials, and Supplemental Materials (including additional exploratory analyses and Study 1 replication) can be accessed at https://osf.io/akc9q/overview.

We determined our sample sizes based on effect sizes reported in Lin et al. [14], who found correlations ranging from *r* = .18 to.38 between collectivism and meaning-making. We used these estimates to conduct our power analyses. Power analyses revealed that we needed about 270 participants to detect the main collectivism-meaning relationship (RQ1) with standard parameters (α = .05, power = .80), and large samples of more than 1,200 participants to be adequately powered for moderation effects tests in RQ2-RQ4. Our sample sizes across studies (Study 1: *n* = 404; Study 2: *n* = 385; Study 3: *n* = 384) exceeded the minimum threshold for detecting the primary correlation effect; we address the underpowered moderator analysis by examining the pooled data after reporting results by study.

#### Analytic approach.

We used null hypothesis significance testing, including correlation analyses, linear regression models, and independent samples t-tests as appropriate for each research question. For RQ1 (association between collectivism and meaning-making), we reported Pearson’s correlation coefficients with *p*-values. For RQ2 (group membership as moderator), we used mixed-effects regression models with collectivism, communicator group membership, and their interaction as predictors. For RQ3 (motivation to seek meaning as moderator), we compared correlation coefficients across Instruction Conditions using 95% confidence intervals and conducted regression analyses with Condition, collectivism, and their interaction. For RQ4 (depth of processing as moderator), we tested interactions between collectivism and memory measures (content and source recall). When comparing correlation coefficients between Conditions or groups, we considered differences statistically significant when 95% confidence intervals (CIs) for these differences excluded zero.

For null findings (95% CIs include 0), we conducted follow-up Bayesian analyses using Bayes Factors (BF₁₀) to quantify evidence for the absence of effects, considering BF₁₀ values below 1/3 as substantial evidence.

We used R Statistical Software version 4.2.1 [69]. Specifically, tidyverse [70] for data wrangling and visualization, plyr [71] for data aggregation, lme4 [72] for mixed-effects modeling, cocor [73] for comparing two correlations using Zou’s [74] confidence interval, and BayesFactor [75] for Bayesian analyses.

## Pre-test and pilot study

Our memory task was based on prior studies, including Turk and colleagues [76], and Jeon and colleagues [57], as reviewed by Dunn [77]. Our goal with the pre-test and the pilot study was threefold. First, to create a bank of statements equated on level of meaningfulness from the full set of machine-constructed statements from Lin, Zhang, and Oyserman, who created statements with metaphor-like structure (i.e., an abstract construct was randomly paired with a concrete one) [14]. To attain this goal, we conducted a pretest in which undergraduates saw the 60 statements (e.g., “Family is honey”), one per screen, and rated each from 1 = *Completely Meaningless* to 7 = *Completely Meaningful*. We used these responses to categorize the average meaningfulness of the statements as low (2.3 to 3.5; *n* = 22 statements), medium (3.51 to 4.5; *n* = 26 statements), or high (4.51 to 5.6; *n* = 14 statements). For the pilot, we randomly drew nine statements at each level of meaningfulness (27 in total). Our second and third goals were to replicate the association between collectivism and meaning-making reported by Lin and colleagues [14] and verify whether this association is moderated by awareness of group membership by collecting data from a new undergraduate sample in our pilot.

### Pilot procedure

**Fig 2:**
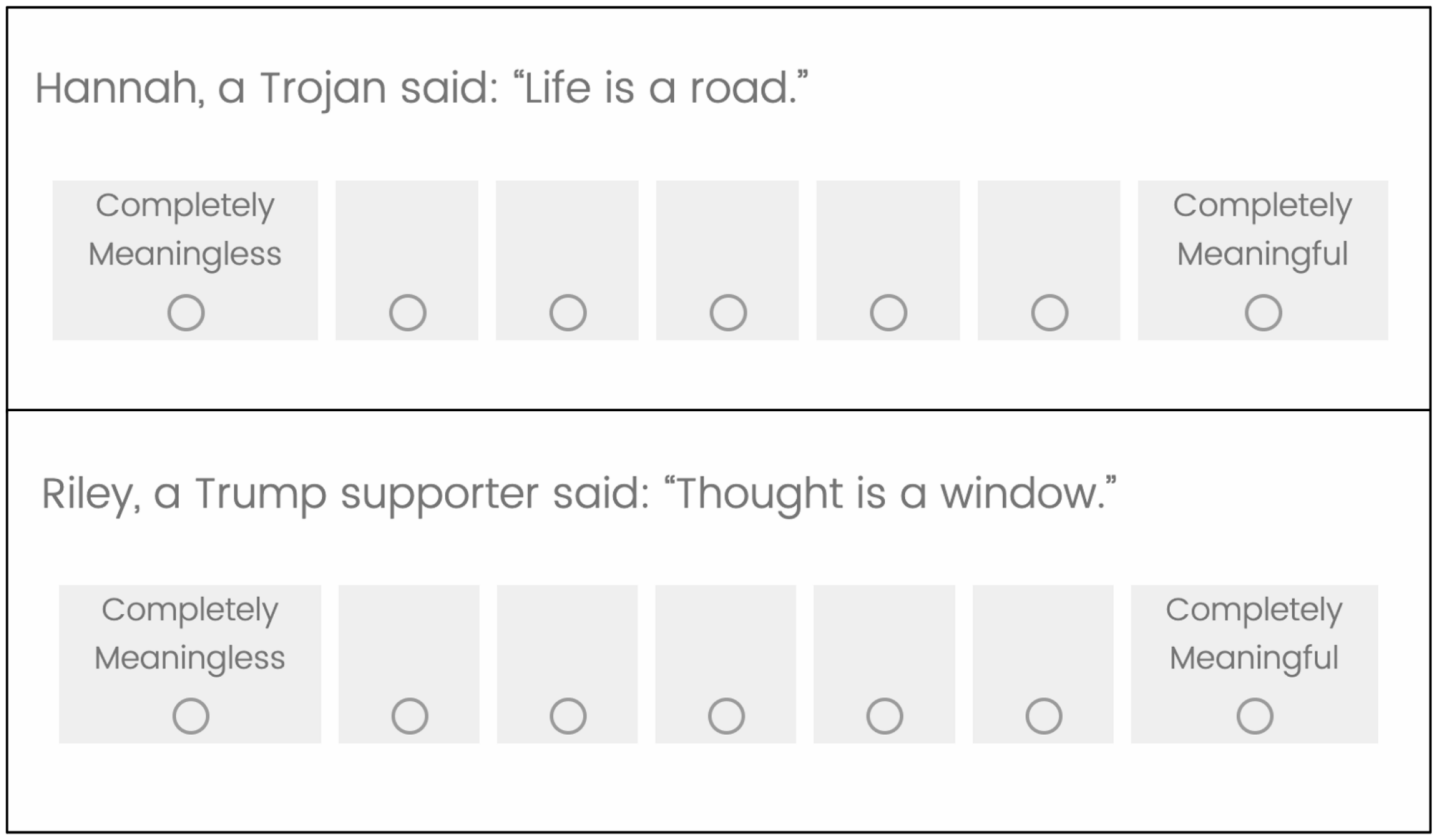
Sample Screens (Top panel Pilot Study and Study 1, Bottom Panel Studies 2 and 3).

The study was programmed in five blocks. Block one was a rating task. Participants read, “Next, you’ll see some statements, some by Trojans from USC and others by Bruins from UCLA.” They then saw 18 statements, one per screen, linked to a first name and a group. An example is in Fig 2, top panel. Each statement remained on the screen for seven seconds while participants rapidly rated meaningfulness from 1 = *Completely Meaningless* to 7 = *Completely Meaningful*. Half of the statements were randomly assigned to a USC Trojan, the other half to the cross-town rival, the UCLA Bruins. First names were selected from the most common American children’s names in 2004, when most participants were born [78]. We chose seven seconds as five seconds is considered a minimum for retention of specific words [79].

Block two was a filler block (a temporal space between the rating and the surprise recall tasks). They read: “Thinking about your college years, how important to you is being a Trojan and graduating from USC?” (1 = *Not Central to Who I Am*, 7 = *Very Central to Who I Am*) and “Imagining your future self, how important do you think being part of the Trojan family will be for your future self?” (1 = *Very Unimportant*, 7 = *Very Important*).

Block three was a surprise memory task. Participants read, “Please read the next set of statements and tell us if you have seen it before or if it is new.” Then they saw 27 statements, one per screen in randomized order (the 18 already seen ones and nine new ones equated on meaningfulness, taken from the pretest). Participants indicated “old” (seen before) or “new” (not seen before) for each statement and specified if the “old” statement came from a Trojan, Bruin, or if they were not sure who said it.

Block four was the collectivism block. Participants rated the 6-item Oyserman [1] Collectivism Scale (α = .69) from 1 = *Strongly Disagree* to 7 = *Strongly Agree*. The six items in the scale were: In general, I accept the decisions made by my group; When I try to understand an event, the first thing that I consider is its implications for my group -- the people who I care about; It often happens that the interests of my group coincide with my own interests; Whatever is good for my group is good for me; If you know what groups I belong to, you know who I am; I tried to understand the needs and wants of my group and act to fulfill them.

Block five was the demographics block. Participants rated from 1 = *Not At All* to 7 = *Very Much* how much they identified as (1) a Trojan and (2) a Bruin, reported age, gender, and ethnicity for sample description.

### Results

Descriptively, as the first column in Table 1 reveals, students scored slightly above the midpoint on collectivism (*M* = 4.53, *SD* = 0.80). Supporting our first research goal of replicating Lin et al.‘s [14] findings, collectivism correlated positively with meaningfulness ratings, *r*(203) =.17, *p* = .016. When examining the potential moderating role of group membership, we found a significant relationship between collectivism and statements from an in-group member, *r*(203) =.20, *p* = .005, not for statements from an out-group member, *r*(203) =.12, *p* = .093. This pattern provided initial evidence that group membership might influence the collectivism-meaning relationship, though the 95% confidence interval for this difference included zero, [−.005,.13], suggesting caution in interpretation.

**Table 1:** Study Source, Size, and Descriptive Information (Mean, Standard Deviation).

Measure	Study, Sample, *N,* Mean, Standard Deviation					
	Pilot	1	2		3	
	Students	Students	Republicans	Democrats	Republicans	Democrats
	*n* = 205	*n* = 405	*n* = 193	*n* = 192	*n* = 194	*n* = 190
Collectivism Scale	4.53 (0.80)	4.57 (0.84)	4.82 (1.01)	4.71 (0.80)	4.84 (1.05)	4.73 (0.85)
Meaningfulness Ratings	3.54 (1.17)	3.65 (1.09)	3.37 (1.27)	2.86 (1.20)	3.60 (1.37)	3.19 (1.07)
Content Recall (Corrected Hit Rates)	.42 (.28)	.48 (.25)	.34 (.25)	.41 (.25)	.34 (.26)	.45 (.23)
Source Recall (Attribution Accuracy)	.26 (.19)	.28 (.20)	.31 (.21)	.28 (.22)	.33 (.21)	.31 (.21)

*Note*: Collectivism and meaningfulness ratings were measured on a 7-point scale (collectivism 1 = *strongly disagree* to 7 = *strongly agree;* meaningfulness 1 = *completely meaningless* to 7 = *completely meaningful*). Content recall is the corrected hit rate (hits minus false alarms). Source recall is source attribution accuracy (correct identification of in-group/out-group communicators).

Following the standard protocol [57], we examined processing depth as content (corrected hit rates) and source recall (the proportion of correctly recognized old statements accurately attributed to an in-group or out-group source, counting “not sure” responses as incorrect). We computed corrected hit rates by subtracting false alarm rates (new statements incorrectly identified as old) from hit rates (old statements correctly identified as old). Content (*M* = .48, *SD* = .25) and source recall (*M* = .28, *SD* = .20) rates were comparable to similar surprise recall paradigms [57], suggesting the task was challenging, indicating adequate sensitivity for our subsequent studies (see Table 1).

The pilot successfully established our methodological approach and provided preliminary support for RQ1 (collectivism is associated with finding meaning in ambiguous statements). We built on the pilot in Study 1 to formally test our research questions.

## Study 1

We used the materials developed in the pre-test and pilot to test RQs 1–4.

### Procedure

There were six blocks. At block one, we randomly assigned participants to one of two instruction sets (seek meaning, seek accuracy). The seek meaning Condition instructed: “Imagine you are interning at the USC counseling service. As part of your training, you must focus on what people mean.” The seek accuracy Condition instructed: “Imagine you are interning at the Daily Trojan. As part of your training, you must accurately report what people say.” Blocks two to six followed the pilot: Rating task at Block two (Fig 2, top panel), filler at Block three; memory task at Block four; and collectivism scale (α = .75; item-level descriptives and correlations in S4 Table) at Block five.

### Results and discussion

**Preliminary Analysis**: Participants identified more with their university mascot (Trojans) than with the rival mascot (Bruins), *p* < .001, validating our in-group/out-group distinction. See Supplemental Materials for descriptive statistics and detailed analyses.

**RQ1:** Supporting RQ1, collectivism score positively correlated with meaningfulness ratings, *r*(402) =.17, *p* < .001.

**RQ2:** To test whether group membership moderates the relationship between collectivism and meaning-making, we conducted a mixed-effects regression analysis accounting for repeated measurements from each participant. We found significant main effects of collectivism (*b* = 0.27, 95% CI [.13,.40], *p* < .001) and communicator group membership (*b* = −0.39, 95% CI [.47, −.31], *p* < .001). The collectivism by group membership interaction, *b* = −0.09, 95% CI [.19,.00], *p* = .056, approached significance. Simple slopes analysis revealed that collectivism significantly predicted meaning ratings when statements came from in-group (*b* = 0.27, 95% CI [.13,.40], p < .001) and out-group members (*b* = 0.18, 95% CI [.04,.31], *p* = .014), suggesting that collectivism predicts meaning-making regardless of message source. We provide model fit statistics for all mixed-effects models and descriptive information on Meaning scores by Group membership in Supplemental Materials, S5 Table, and S6 Table.

**RQ3:** We tested whether meaning-seeking instructions moderate the relationship between collectivism and meaning-making with a regression analysis with three predictors: collectivism (*b* = 0.30, 95% CI [.12,.48], *p* = .001), Condition (*b* = −0.08, 95% CI [−.29,.13], *p* = .473), and their interaction (*b* = −0.16, 95% CI [−.41,.09], *p* = .196). Follow-up analyses revealed that collectivism scores significantly correlated with meaningfulness ratings among participants randomly assigned to the Accuracy Condition, *r*(203) =.23, 95% CI [.09,.35], *p* = .001 (Meaning Condition *r*(197) =.11, 95% CI [−.03,.24], *p* = .158.) However, the 95% confidence interval of the difference in correlations by Condition included zero (CI _*r* difference_ [−.07,.31]) and a Bayesian analysis yielded a BF₁₀ = 0.33, weak evidence that the correlations differ, leading us to infer that our subtle Condition manipulation may not be sufficient to moderate the relationship between collectivism and meaning-making.

**RQ4:** To examine whether depth of processing moderates the relationship between collectivism and meaning-making, we conducted two regression-based moderation analyses, one focusing on content recall (corrected hit rates) and the other on source recall (source attribution accuracy). For content recall, a regression analysis revealed significant main effects of collectivism (*b* = 0.32, 95% CI [.08,.55], *p* = .009) and content recall (corrected hit rates *b* = −0.78, 95% CI [−1.19, −0.36], *p* < .001), but no significant interaction (*b* = −0.15, 95% CI [−.60, 0.30], *p* = .509). A Bayesian analysis of this interaction yielded BF₁₀ = 0.24, providing substantial evidence for the null hypothesis (content recall does not moderate the collectivism-meaning relationship). The source recall regression revealed neither main (collectivism *b* = 0.16, 95% CI [−.04,.36], *p* = .122, source recall accuracy *b* = 0.37, 95% CI [−.17,.91], *p* = .177), nor interaction (*b* = 0.20, 95% CI [−.37,.77], *p* = .492) effects. A Bayesian analysis yielded BF₁₀ = 0.22 for this interaction, again providing substantial evidence against a moderating effect of source recall. In sum, traditional null hypothesis testing and Bayesian analyses converge in suggesting that processing depth, as measured by content and source recall, does not moderate the relationship between collectivism and meaning-making in the university context. Detailed information on group differences in recall for statement content and source can be found in S7 and S8 Tables. A follow-up using similar materials and procedures with a new sample of undergraduates (*N* = 386) largely replicated these patterns, with some additional findings reported in Supplemental Materials.

## Study 2

### Procedure

We collected data during the week before the 2024 U.S. Presidential election in an 8-minute online experiment on Prolific for $2.14 (following the 2024 California $16.00 hourly minimum wage. We maintained the Study 1 six-block structure.

In Block 1, participants were randomly assigned to an instruction (seek meaning or seek accuracy). Meaning Condition participants read, “Imagine you are tasked with being a moderator for a social media site you care about. As a moderator, your task is to consider what people are trying to communicate when they say things. That may mean reading between the lines and inferring what that might be.” In contrast, Accuracy Condition participants read, “Imagine you are tasked with being a moderator for a social media site you care about. As a moderator, your task is to consider how accurate people are when they say things. That may mean verifying and checking what they say.”

In Block 2, participants saw the ambiguous statements, one per screen, in a randomized order and again rated each for meaningfulness on a 7-point scale (1 = *Completely Meaningless* to 7 = *Completely Meaningful*). Half of the statements were attributed to a Trump supporter and half to a Harris supporter. Study 2 did not have an auto-advance time constraint. Participants advanced to the next statement by pressing “next” when ready. We note that the time spent was comparable to the amount of time we provided in Study 1 (auto-advance after seven seconds); in Study 2, on average, participants spent just under six seconds per statement. To control for potential statement effects, we randomized participants to one of two counterbalanced versions of the statements so that across versions, each statement was equally likely to be paired with an in-group communicator or an out-group communicator. Sample screens are shown in Fig 2.

The filler questions were adapted to the political context:(1) “Thinking about your identity, how important to you is being a Harris supporter?” (2) “Thinking about your identity, how important to you is being a Trump supporter?” (3) “Imagining your future self, how important do you think being a Harris supporter now will be for your future self?” and (4) “Imagining your future self, how important do you think being a Trump supporter now will be for your future self?”

In Blocks 4, 5, and 6, participants completed the surprise memory task, the collectivism scale (α = .79; item-level descriptives and correlations in S4 Table), and demographic questions, in that order.

### Results

**Preliminary Analyses:** Republicans identified significantly more with a “Trump supporter” than a “Harris supporter,” and Democrats identified with a “Harris supporter” than a “Trump supporter” (all *p*s < .001, see Supplemental Materials for detailed analyses). We did not find differences between Republicans (*M* = 4.82, *SD* = 1.01) and Democrats (*M* = 4.71, *SD* = 0.80) in collectivism scores, *t*(385.56) = 1.25, *p* = .211, or in iden*t*ifying with the in-group (Republicans: *M* = 4.83, *SD* = 1.80; Democrats: *M* = 4.70, *SD* = 1.74), *t*(382.69) = 0.74, *p* = .457.

**RQ1:** Supporting RQ1, collectivism was positively correlated with meaningfulness ratings, *r*(383) =.19, 95% CI[.10,.29], *p* < .001.

**RQ2:** We conducted a mixed-effects regression analysis accounting for repeated measurements to test whether group membership moderates the collectivism-meaning-making relationship, revealing main and interaction effects (collectivism *b* = 0.33, 95% CI[.19,.48], *p* < .001; communicator group membership *b* = −0.35, 95% CI[−.44, −.27], *p* < .001; collectivism x group membership interaction *b* = −0.13, 95% CI[−.23, −.04], *p* = .007). Simple slopes analyses revealed that the collectivism-meaning-making relationship was stronger for statements from the in-group (*b* = 0.33, 95% CI [.19,.48], *p* < .001), compared to the out-group (*b* = 0.20, 95% CI [.06,.34], *p* = .006). We provide model fit statistics for all mixed-effects models and descriptive information (including separately for Democrats and Republicans) in Supplemental Materials, S5 Table and S6 Table.

**RQ3:** We tested whether random assignment to the seek meaning vs. seek accuracy instruction moderates the relationship between collectivism and meaning-making by examining the interaction between collectivism and Instruction Condition (Meaning vs. Accuracy). A regression analysis revealed a significant main effect of collectivism (*b* = 0.26, 95% CI [.08,.45], *p* = .005, Condition *b* = 0.18, 95% CI [−.07,.43], *p* = .161, collectivism x Instruction Condition interaction *b* = 0.03, 95% CI [−.25,.30], *p* = .852). The Bayesian analyses (BF_10_ = 0.18) provided substantial evidence against moderating effects of the subtle instruction to seek meaning (vs. accuracy) on the collectivism-meaning-making relationship.

**RQ4:** In two regression equations, we analyzed whether variance in depth of processing, operationalized as memory for content and for source, moderates the relationship between collectivism and meaning-making. Both regressions yielded a main effect for collectivism. The memory for source regression also showed a main effect of source recall. That is, people who rated a statement as more meaningful were both higher in collectivism and incidentally processed who made the statement. In the memory for content regression: collectivism *b* = 0.46, 95% CI [.22,.70], *p* < .001, content recall *b* = −0.10, 95% CI [−.59,.40], *p* = .704), collectivism by content recall interaction (*b* = −0.53, 95% CI [−1.06,.00], *p* = .051); in the memory for source regression: collectivism *b* = 0.16, 95% CI [−.08,.40], *p* = .198), source recall *b* = 1.19, 95% CI [.62, 1.76], *p* < .001), collectivism by source recall interaction *b* = 0.19, 95% CI [−.45,.83], *p* = .563). Though not significant, Bayesian analyses provided support for a collectivism by content recall interaction, BF₁₀ = 1.19, implying that people higher in collectivism who process more deeply rate statements as more meaningful. In contrast, while better recall for source is directly associated with meaning ratings, it does not moderate the relationship between collectivism and meaning-making (Bayesian analyses provided substantial evidence against a moderating role of source recall for the overall sample, BF₁₀ = 0.21).

## Study 3

### Procedure

We collected data two months into Donald Trump’s (Republican) term as the 47th president of the United States. Study 3 maintained the six-block structure of Study 2. Blocks 1 (randomization), 4 (the collectivism scale, α = .82; item-level descriptives and correlations in S4 Table), and 5 (demographic questions) were identical to Study 2; in Blocks 2 (rating task) and 3 (filler), statements were attributed to a “Trump supporter” or a “Democrat” given that Harris supporter was likely no longer a salient category. In Block 6 (demographics), we added two items (1 = *Not At All* to 7 = *Very Much*) to assess trust in (1) Trump supporters and (2) Democrats.

### Results

**Preliminary Analyses:** Republicans identified significantly more with Trump supporters than Democrats, and Democrats identified more with Democrats than Trump supporters (all *p*s < .001, see Supplemental Materials for detailed analyses).

Republicans (*M* = 4.84, *SD* = 1.05) and Democrats (*M* = 4.73, *SD* = 0.85) did not differ in collectivism, *t*(368.94) = 1.15, *p* = .253, or in-group identification (Republicans *M* = 5.28, *SD* = 1.76; Democrats *M* = 5.24, *SD* = 1.71, *t*(381.99) = 0.23, *p* = .815). Republicans trusted Republicans (*M* = 5.04, *SD* = 1.53) more than Democrats (*M* = 2.52, *SD* = 1.40), *t*(193) = 16.06, *p* < .001, and Democrats trusted Democrats (*M* = 4.45, *SD* = 1.38) more Republicans (*M* = 1.46, *SD* = 0.78), *t*(189) = 26.95, *p* < .001. Republicans trusted Republicans more than Democrats trusted Democrats, *t*(379.47) = 3.96, *p* < .001, and also trus*t*ed Democrats more than Democrats trusted Republicans, *t*(304.17) = 9.23, *p* < .001. As detailed in Supplemental Materials, exploratory analyses with trust as an additional moderator in the RQ1-RQ4 models did not reveal a consistent trust effect.

Regarding our two depth of processing indicators, Democrats scored higher than Republicans in content recall, *t*(378.6) = 4.22, *p* < .001; the groups did not differ in source recall, *t*(381.92) = 0.83, *p* = .406. See Table 1 for means and s*t*andard deviations.

**RQ1:** Supporting RQ1, collectivism was positively correlated with meaningfulness ratings *r*(382) =.18, 95% CI [.08,.27], *p* < .001

**RQ2:** A mixed-effects regression model accounting for repeated measurements revealed significant main effects of collectivism (*b* = 0.25, 95% CI[.11,.38], *p* < .001) and communicator group membership (*b* = −0.23, 95% CI[−.31, −.15], *p* < .001; interaction *b* = −0.06, 95% CI[−.15, 02], *p* = .151).

**RQ3:** Regression analysis revealed main effects of collectivism (*b* = 0.28, 95% CI[.11,.46], *p* = .001) and Instruction Condition (*b* = 0.20, 95% CI[−.04,.45], *p* = .106; collectivism x Condition *b* = −0.13, 95% CI[−.39,.16], *p* = .313). The follow-up Bayesian analysis (BF₁₀ = 0.28) provided substantial evidence for the null hypothesis that the Instruction Condition moderates the collectivism-meaning relationship.

**RQ4:** The memory regressions revealed main effects. For content regression analysis: collectivism (*b* = 0.24, 95% CI[.01,.48], *p* = .043) and memory for content (*b* = 0.11, 95% CI[−.38,.60], *p* = .651, not their interaction *b* = −0.03, 95% CI[−.58,.52], *p* = .911). For message source regression analysis: collectivism (*b* = 0.09, 95% CI[−.12,.31], *p* = .390) and memory for message source (*b* = 1.27, 95% CI[.69, 1.86], *p* < .001; collectivism by source recall interaction *b* = 0.33, 95% CI[−.21,.87], *p* = .226). Yet the follow-up Bayesian analysis suggested inconclusive evidence for the moderation effects of deeper processing on the collectivism-meaning relationship (memory for content BF₁₀ = 0.20; memory for source BF₁₀ = 0.36).

## Pooled data analysis

We combined data across Studies 1–3 (*N* = 1,174) to test our moderation predictions with better power and to explore possible effects of political group membership (our EQ). The pooled data set included a variable for Study as a fixed effect, given the different contexts (university affiliation vs. political identity) and time (before and after the Presidential Election), so that each of the potential sources of variance could be controlled. We used multilevel models with random intercepts for Study as a robustness check to account for the nested structure of observations within studies. Complete statistical details for the pooled analyses are provided in Supplemental S9 Table, with multilevel modeling results reported in S10 Table. Additional robustness checks, including leave-one-study-out sensitivity analyses and post-hoc power analyses, are reported in Supplemental S11 and S12 Tables. We followed up with EQ analysis of Study 2 and 3 data combined (*N* = 769) to explore if effects differ between Democrats and Republicans.

**RQ1:** Supporting RQ1, collectivism predicted finding meaning in ambiguous statements, *r*(1171) =.16, *p* < .001, including when adding study as a control, *b* = 0.24, 95% CI [.16,.31], *p* < .001. Adding study significantly improved model fit, χ²(2) = 48.85, *p* < .001, with participants in the political context finding less meaning than those in the university mascot context (Study 2 *b* = −0.60, 95% CI [−.76, −.43], *p* < .001; Study 3 *b* = −0.32, 95% CI [−.48, −.15], *p* < .001). As a robustness check, the multilevel modeling results with random intercepts for Study remained highly consistent (*b* = 0.24, 95% CI [.16,.31], *p* < .001). Thus, though processing messages in a political context reduced overall meaning-making compared to processing messages in a less fraught university context, people higher in collectivism found more meaning in ambiguous statements across contexts.

**RQ2**: Supporting RQ2, group membership significantly moderated the effect of collectivism on finding meaning in a mixed-effects analysis, *b* = −0.09, 95% CI [−.14, −.03], *p* = .002. Multilevel models with random intercepts for Study confirmed this moderation (*b* = −0.09, 95% CI [−.14, −.04], *p* = .002). Follow-up simple slopes analysis revealed that collectivism was associated with finding meaning in in-group (*b* = 0.28, 95% CI [.20,.36], *p* < .001) and out-group (*b* = 0.20, 95% CI [.12,.28], *p* < .001) members’ statements, but the effect of collectivism was stronger for in-group than out-group. Including study controls significantly improved model fit, χ²(2) = 42.36, *p* < .001. Thus, messages from in-group sources are preferred, as revealed in the significant moderation, even though collectivism is associated with finding more meaning even in the ambiguous communication of out-group members.

**RQ3:** Regarding RQ3, we did not find evidence that subtle instruction to seek meaning rather than accuracy moderated the effect of collectivism on finding meaning in ambiguous statements, *b* = −0.07, 95% CI [−.22,.08], *p* = .342. The multilevel modeling results consistently did not support the moderation effect (*b* = −0.07, 95% CI [−.22,.08], *p* = .344). Follow-up Bayesian analysis provided substantial evidence against moderation, BF₁₀ = 0.13. As in the by-study analyses, we found a main effect of collectivism, *b* = 0.27, 95% CI [.17,.38], *p* < .001, and no main effect of Instruction Condition, *b* = 0.10, 95% CI [−.04,.23], *p* = .164. Study controls again significantly improved fit, χ²(2) = 49.20, *p* < .001.

**RQ4:** RQ4 was not supported; we did not find evidence that processing depth moderated the effect of collectivism on finding meaning, whether we tested this as recalling message content (*b* = −0.18, 95% CI [−.47,.11], *p* = .232), or message source (*b* = 0.29, 95% CI [−.04,.62], *p* = .086). The follow-up analyses revealed that the multilevel modeling result remained consistent with non-significant moderation effects (content *b* = −0.17, 95% CI [−.46,.12], *p* = .240; source *b* = 0.29, 95% CI [−.04,.62], *p* = .087) and moderate Bayesian evidence against moderation (content BF₁₀ = 0.14, source BF₁₀ = 0.31). As in the by-study analyses, we found a main effect of collectivism, and, as in some of the by-study analyses, a main effect of source recall (see Supplemental S8 Table). Study controls again significantly improved fit (content χ²(2) = 53.11, *p* < .001; source χ²(2) = 53.54, *p* < .001).

**EQ:** To explore if RQ1 to RQ4 effects were consistent for Republicans and Democrats, we combined Study 2 and Study 3 data (*N* = 769). Regarding RQ1, the positive association between collectivism and statement meaningfulness ratings was consistent for both groups (Republicans *r*(385) =.21, 95% CI [.03,.30], *p* < .001; Democrats *r*(380) =.14, *p* = .008). The strength of this association did not significantly differ between Republicans and Democrats (95% CI r difference [−.21,.07]). Bayesian analysis comparing models with and without the collectivism x party affiliation interaction provided substantial evidence supporting the null hypothesis of no significant difference in the collectivism-meaning-making relationship between Republicans and Democrats (pooled BF₁₀ = 0.15).

Regarding RQ2, the mixed effect model showed significant main effects of collectivism (Democrats: *b* = 0.27, 95% CI [.13,.42], *p* < .001; Republicans: *b* = 0.29, 95%% CI [.15,.42], *p* < .001) and communicator group membership (Democrats *b =* −0.33, 95% CI [−.41, −.25], *p* < .001; Republican *b* = −0.25, 95% CI [−.34, −.16], *p* < .001). The collectivism × group membership interaction was significant for Democrats (*b* = −0.17, 95% CI [−.26, −.08], *p* < .001), not Republicans (*b =* −0.05, 95% CI [−.13,.04], *p* = .299). Simple slopes analyses indicated that among Democrats, collectivism positively predicted finding meaning in statements from the in-group (*b* = 0.27, 95% CI [.13,.42], *p* < .001) not from the out-group (*b* = 0.10, 95% CI [−.04,.25], *p* = .167). However, among Republicans, collectivism positively predicted finding meaning in statements from both the in-group (*b* = 0.29, 95% CI [.15,.42], *p* < .001) and the out-group (*b* = 0.24, 95% CI [.11,.37], *p* < .001). The follow-up Bayesian model comparison provided evidence against including a three-way interaction (BF₁₀ = 0.17), suggesting that the pattern did not reliably differ between Republicans and Democrats.

Regarding RQ3, we found significant main effects of collectivism and Instruction Condition but no collectivism by Condition interaction among Republicans and Democrats (Collectivism: Democrats: *b* = 0.12, 95% CI [−.07,.31], *p* = .222; Republicans *b* = 0.34, 95% CI [.17,.50], *p* < .001; Instruction Condition: Democrats *b* = 0.24, 95% CI [.01,.46], *p* = .044; Republicans: *b* = 0.14, 95% CI [−.12,.40], *p* = .290; collectivism x Condition; Democrats: *b* = 0.16, 95% CI [−.12,.44], *p* = .259; Republicans: *b* = −0.17, 95% CI [−.43,.08], *p* = .182). The Bayesian model comparison provided evidence against including a three-way interaction (BF₁₀ = 0.68).

Regarding RQ4, we again did not find evidence of a moderating effect of depth of processing operationalized as memory for statement content and memory for communicator group membership. In the content recall regression (Collectivism: Republicans *b =* 0.37, 95% CI [.16,.59], *p* < .001; Democrats *b =* 0.17, 95% CI [−.12,.46], *p* = .250; Content recall: Republicans *b =* −0.07, 95% CI [−.59,.44], *p* = .781; Democrats *b =* 0.47, 95% CI [−.01,.94], *p* = .055; Collectivism x Content Recall: Republicans *b =* −0.34, 95% CI [−.86,.18], *p* = .203; Democrats *b =* 0.07, 95% CI [−.52,.66], *p* = .819). In the source recall regression: Collectivism (Republicans: *b =* 0.06, 95% CI [−.15,.28], *p =* .578; Democrats (*b =* 0.24, 95% CI [.01,.48], *p* = .044; Source Recall: Republicans *b =* 1.42, 95% CI [0.82, 2.03], *p <* .001; Democrats *b =* 1.05, 95% CI [0.53, 1.58], *p <* .001; Collectivism x Source Recall: Republicans: *b* = 0.48, 95% CI [−.07, 1.02], *p* = .085; Democrats: *b =* −0.26, 95% CI [−.89,.37], *p* = .413). The Bayesian model comparison (content recall BF₁₀ = 0.33; source recall BF₁₀ = 0.77) suggests support for the null effect that the political affiliation did not moderate the collectivism and source recall relationship.

## General discussion

Across three studies, we found consistent evidence that collectivism is positively associated with a tendency to find meaning in ambiguous statements, supporting our first research question. This foundational relationship remained stable across various group contexts, including university affiliation and political identity, and a pooled analysis of all studies. Our research questions two to four explored three theory-central moderators: group membership, contextual support for finding meaning, and depth of processing.

Supporting our second research question, group membership of the communicator moderates this relationship in context-dependent ways. Our pooled analysis confirmed that more meaning is inferred from in-group communications and less meaning was inferred overall in political compared to university settings. The effect of in-group membership was particularly pronounced for Democrats.

To examine our third research question (whether collectivism increases the likelihood of finding meaning because it triggers searching for meaning), we subtly manipulated whether attention was directed to search for statement meaning or accuracy. Our pooled analyses did not support this inference, even though we found support in some individual studies. The relationship between collectivism and meaning-making may operate as a stable individual difference, and disrupting the process may require a stronger contextual cue than we provided.

Regarding our fourth research question, we measured incidental memory for content and source to address the possibility that people higher in collectivism automatically process statements more deeply (content recall) and consider the messenger (source recall). We do not find support in pooled analyses. Potentially, other measures of processing depth, such as concurrent generation of reasons for finding meaning, might better address this theory-relevant mechanism.

### Theoretical implications for collectivism and meaning-making

Our results connect with theory in five areas: collectivism (research exploring the connection between collectivism and finding meaning even in ambiguous or empty claims); culture-as-situated cognition (research highlighting the situated effects of collectivism on reasoning); communication (research on how people construe meaning); motivated reasoning (research examining the relationship between identity and meaning-making); and depth of processing research. We briefly consider each next.

First, earlier research documents that collectivism is associated with finding meaning even in ambiguous claims, with even empty claims by triggering an active construction of meaning [14]. Follow-up studies show the association between collectivism and finding meaning in humor, an unstructured form of communication [80]. We replicate and extend the results of Lin, Zhang, and Oyserman [14] in two ways: First, we highlight important nuances in how this relationship operates across different contexts and second, we highlight how this relationship is fundamentally shaped by the salience of group membership, the specific nature of group identity, and the political context.

Second, our results are congruent with a situated social-cognition theory of culture by highlighting that collectivism operates as a dynamic, context-sensitive mindset whose effects vary based on social identity activation and environmental demands [2,3]. Collectivists do not merely passively accept information but actively engage with it as a fundamentally social act [37,38]. Moreover, our results, of differential patterns in university and political contexts, support a culture-as-situated cognition perspective [2,3]. Being a graduate of a particular university is an enduring group membership, but it is also enduringly nested in another group membership -- the group of college graduates (or in the current sample, the group of elite college graduates). In university settings, collectivistic meaning-making operated across group boundaries, suggesting that when group distinctions are less consequential, collectivistic tendencies manifest as a general orientation toward finding social meaning. In contrast, while being a member of a political party is at least in principle malleable, an identity that can be swapped for another (a member of a different political party or no political party), at least at the time of our data collection, party membership was very stable [81] (and we focused on people who voted, the more active group members). In addition to its stability, party membership is likely experienced as a higher-stakes inter-group context (especially at the time of our data collection, before and after the election of Donald Trump, a Republican, as the 47th President of the United States). We found that meaning-making was selectively oriented toward in-group among collectivists, particularly among Democrats, the underdogs rather than the elected majority in this higher stakes setting.

Third, our results are relevant to communication research. Communication is bi-directional, with receivers adjusting their meaning-making strategies based on social identity factors and communicative contexts [82,83]. Our results demonstrate that this adjustment process varies systematically depending on the stakes involved. In low-stakes university contexts, collectivistic meaning-making operates broadly across sources. In higher-stakes, political contexts, in contrast, collectivistic meaning-making becomes strategically selective, particularly for Democrats, who showed a consistent in-group-only uptick in meaning-making across both periods. While we focused on active construction of meaning from ambiguous information, our results also connect to the sociological research on resonance, which describes messages as being persuasive if they are constructed from culturally meaningful materials [84]. We describe a complementary aspect of persuasiveness, which occurs when collectivists create meaning from ambiguous communication.

Fourth, our results are relevant to theories of identity-protective motivated cognition [61,85] and partisan information processing [60]. These theories predict that cultural orientations adapt to protect valued group identities when those identities become salient and threatened. The selectivity we observed fits research in this area: people process in-group information differently [54], and this increases their vulnerability to accepting questionable claims [86–88]. Our findings suggest that collectivistic tendencies amplify this differential processing when group membership shifts from symbolic (university affiliation) to consequential (political identity), potentially increasing susceptibility to partisan misinformation through active meaning construction rather than analytical evaluation.

Our mixed findings regarding motivation to seek meaning provide potential insights into the nature of collectivistic meaning-making. The isolated instances in which instruction to seek meaning eliminated the collectivism-meaning relationship (Study 1 and Republicans in Study 3) suggest that collectivistic meaning-making may operate through spontaneous meaning-seeking procedures that become redundant when externally prompted. Inconsistent results across contexts and the resistance to instruction effects among Democrats overall, as well as among both groups during the pre-election period, suggest that strong group identities may override typical cognitive procedures, which is congruent with identity-protection theories [60,61]. This implies that collectivistic meaning-making becomes more automatic and less controllable when political identities are highly salient.

Lastly, our results for depth of processing suggest important insights about how collectivism operates in context. Rather than operating through enhanced analytical processing, as suggested by levels of processing theory [48], collectivistic meaning-making appears to function through social-attentional mechanisms independent of the depth of systematic processing. The finding that source recall predicted meaning-making independently of collectivistic tendencies in political contexts supports research suggesting differential attention to source versus content information in political communication [66]. These results align with theories of collectivism that predict that when group identities are salient, collectivists prioritize social and relational aspects of communication over detailed content analysis [34,36].

### Limitations and future directions

We focus on limitations and future directions in our context variable, our methods of manipulation and measurement, and our sample sizes and sources. First, regarding context, we examined pre-election and post-inauguration political contexts but did not measure context as a continuous variable. Future research could include more contexts or obtain within-person context effects with a diary or ecological momentary assessment method. The heightened polarization of the pre-election period and the transitional nature of the immediate post-inauguration period represent particular moments with unique characteristics. Second, we used a subtle manipulation of propensity to seek meaning to test the prediction that collectivism is associated with meaning-making because it triggers seeking meaning. Future research could test alternative manipulations, or more heavy-handed ones, to test whether this process can be activated through other means or stronger cues. It is possible that our null effects were due to an overly subtle manipulation. Moreover, other collectivism-relevant triggers of meaning making could be tested, including making accessible the likelihood of future interactions with in-group members, which is another pathway by which collectivism could increase perceived meaning. Similarly, our recognition-based memory task measured content and source recall but may not fully capture the qualitative aspects of how people process and remember information. More nuanced memory measures could provide richer insights into how collectivism shapes the depth and nature of information processing. Our meaning-making measure was based on ambiguous statements designed to be relatively low in meaning. This allowed for a strong test but limited ecological validity. Future research might use experience sampling or ecological momentary assessment to have people rate real messages that they encounter. Other ways of operationalizing depth of processing could be used, for example, coding not just whether people found ambiguous statements meaningful, but the meaning that they drew could be used (e.g., [12,89]. Lastly, exposure times were brief, and our tests of processing focused on incidental recall of features (source, content) that were distinct from the initial task of rating the statement for meaningfulness. Though effects did not change when we allowed people to proceed at their own pace, it is possible that longer exposure or analysis of processing separate from memory would yield stronger effects. Moreover, we measured collectivism with a particular measure. It is possible that alternative measures of collectivism would capture more variance. Our theoretical framework focused on collectivism and processing for meaning. We tested for the effects of group membership. Future research could separately manipulate communicator status and group membership. It is possible that people higher in vertical collectivism [90] would be particularly likely to process for meaning if the status as well as group membership of the communicator was made salient.

Third, our samples represented university students and political partisans, and our sample sizes were adequate for detecting the main collectivism-meaning relationship. While powered in the pooled analyses for detecting moderation effects, at the study level, our samples were underpowered for reliably detecting moderation effects. Hence, future studies could test the stability of significant interactions that emerged in individual studies but not in our pooled analyses, and obtain samples from outside the United States to examine whether the patterns we observed extend to other cultural and political systems with different partisan dynamics.

Despite these limitations, our research provides valuable insights into how collectivism shapes meaning-making across different group contexts and opens several promising avenues for future research. As we noted above, these include within- and between-person analyses of context effects across political transitions to obtain a clearer estimate of within-person and between-person effects of collectivism on meaning-making. Such studies could include ecologically valid messages of varying ambiguity and partisan framing. Another new venue would be to explore how collectivism shapes the processing of corrections and retractions of in-group or out-group members. Effects could be tested with group memberships with higher and lower stakes (e.g., political groups, university alma mater) to test the boundaries of findings.

### Conclusion

We document the complex interplay of collectivism, group identity, and meaning-making in communication processes across different contexts. We demonstrate that collectivism operates not as a fixed cultural trait but through social identities that fundamentally shape how people engage with ambiguous information. When group distinctions are less consequential, collectivistic meaning-making operates broadly; when group distinctions are more consequential, it becomes strategically selective.

## Supporting information

S1 Table Pre-Test & Pilot Study: Detailed Demographics with Categories in the Order Presented to Participants. (DOCX)

S2 Table Study 1 & Study 1 Replication: Detailed Demographics with Categories in the Order Presented to Participants. (DOCX)

S3 Table Studies 2 & 3: Detailed Demographics with Categories in the Order Presented to Participants (Study 3 Gender Options: Male, Female, And Another Description with Textbox After Click). (DOCX)

S4 Table Studies 1–3: Collectivism Item Means, Standard Deviations, and Between-Item Correlations, All Significant at p < .001. Items were 1: In general, I accept the decisions made by my group. 2: When I try to understand an event, the first thing that I consider is its implications for my group -- the people who I care about. 3: It often happens that the interests of my group coincide with my own interests. 4: Whatever is good for my group is good for me. 5: If you know what groups I belong to, you know who I am. 6: I tried to understand the needs and wants of my group and act to fulfill them.
(DOCX)

S5 Table Mixed-Effects Model Fixed Effects, Random Effects Structure, and Model Fit Statistics Where N(obs/grps) Is the Number of Observations and of Level-2 Groups (Participants in This Case). (DOCX)

S6 Table Group Membership Effects Statement Meaningfulness Rating Except for Study 3 Republicans, for Which a Bayesian analysis (BF₁₀ = 0.17) Provided Substantial Evidence for the Null Hypothesis of No Difference In Meaningfulness Ratings Between In-Group and Out-Group Statements. (DOCX)

S7 Table Content Recall, Assessed as Corrected Hit Rates (Hits Minus False Alarms) By Communicator Source, Studies 2 and 3 < .33 Bayes Factors (BF₁₀) Score Indicate Substantial Evidence for the Null Hypothesis of No Difference in Content Recall as a Function Of Group Membership. (DOCX)

S8 Table Source Recall Operationalized As Source Attribution Accuracy (Correct Identification Of The Source Of Communication By Group Membership) By Communicator Source (Studies 2 And 3 Bayes Factors (BF₁₀) Indicated Substantial To Moderate Evidence For The Null Hypothesis Of No Difference In Source Memory Based On Communicator Group Membership). (DOCX)

S9 Table Complete Statistical Results for Pooled Analysis (N = 1,174). ΔR² = change model adding study controls vs. models without control; all models include study as a control variable with Study 1 as the reference category. R²_adj_ not reported for RQ2 mixed-effects model due to the complexity of variance partitioning with random effects. Group: Ingroup = 0, Outgroup = 1. Condition: Accuracy = 0, Meaning = 1. RQ2 Mixed-effects model accounts for repeated measures within participants.
(DOCX)

S10 Table Pooled Analyses Using Multilevel Modeling with Random Intercepts for Study. Note. ICC = the study-level variance proportion after accounting for participant clustering All models included random intercepts for Study. For RQ2, the model also included random intercepts for participants.
(DOCX)

S11 Table Leave-One-Study-Out Sensitivity Analyses for Pooled Data. (DOCX)

S12 Table Post-hoc Power Analysis Computed using two-tailed α = .05. In linear regression models, power was based on increase in explained variance (Δ*R²*) when adding the predictor to the model expressed as Cohen’s *f²*. RQ2 power for the mixed-model interaction estimated using simr::powerSim (*n* = 1,000).
(DOCX)

S1 File Preliminary Analyses: In-Group/Out-Group Validation. (DOCX)

S2 File Study 1 Replication. (DOCX)
