# Supplementary material for: Collectivism and meaning-making: A search for moderators
Source: PLoS One. 2026 Apr 30;21(4):e0346979. doi: 10.1371/journal.pone.0346979 (PMC13132207; doi:10.1371/journal.pone.0346979)
Supplement: S1 Table — (DOCX) [file pone.0346979.s001.docx]

|  | Pre-Test | | Pilot | |
| --- | --- | --- | --- | --- |
|  | Undergraduates (*n* = 99) | | Undergraduates (*n* = 205) | |
|  | *n* | % | *n* | % |
| Gender |  |  |  |  |
| Male | 32 | 32.3 | 68 | 33.2 |
| Female | 63 | 63.6 | 127 | 62.0 |
| Non-Binary | 0 | 0.0 | 5 | 2.4 |
| Transgender | 1 | 1.0 | 1 | 0.5 |
| Choose not to answer | 0 | 0.0 | 4 | 2.0 |
| Race-Ethnicity |  |  |  |  |
| Non-Hispanic White | 16 | 16.2 | 45 | 22.0 |
| Black, African American, Afro-Caribbean | 10 | 10.1 | 9 | 4.4 |
| Latino or Hispanic | 13 | 13.1 | 41 | 20.0 |
| East Asian | 25 | 25.3 | 55 | 26.8 |
| South Asian | 4 | 4.0 | 28 | 13.7 |
| Middle Eastern or Arab | 3 | 3.4 | 7 | 3.4 |
| Native American or Alaskan Native | 0 | 3.0 | 2 | 1.0 |
| Mixed | 13 | 13.1 | 13 | 6.3 |
| Other | 11 | 11.1 | 4 | 2.0 |
