# Supplementary material for: Collectivism and meaning-making: A search for moderators
Source: PLoS One. 2026 Apr 30;21(4):e0346979. doi: 10.1371/journal.pone.0346979 (PMC13132207; doi:10.1371/journal.pone.0346979)
Supplement: S2 Table — (DOCX) [file pone.0346979.s002.docx]

|  | Study 1 | | Study 1 Replication | |
| --- | --- | --- | --- | --- |
|  | Undergraduates (*n* = 405) | | Undergraduates (*n* = 386) | |
|  | *n* | % | *n* | % |
| Gender |  |  |  |  |
| Male | 138 | 34.1 | 128 | 33.2 |
| Female | 259 | 64.0 | 248 | 64.2 |
| Non-Binary | 6 | 1.5 | 7 | 1.8 |
| Transgender | 0 | 0.0 | 0 | 0.0 |
| Choose not to answer | 2 | 0.5 | 3 | 0.8 |
| Race-Ethnicity |  |  |  |  |
| Non-Hispanic White | 80 | 19.8 | 107 | 27.7 |
| Black, African American, Afro-Caribbean | 20 | 4.9 | 13 | 3.4 |
| Latino or Hispanic | 66 | 16.3 | 41 | 10.6 |
| East Asian | 94 | 23.5 | 82 | 21.2 |
| South Asian | 38 | 9.4 | 26 | 6.7 |
| Middle Eastern or Arab | 15 | 3.7 | 22 | 5.7 |
| Native American or Alaskan Native | 0 | 0.0 | 0 | 0.0 |
| Mixed | 30 | 7.4 | 41 | 10.6 |
| Other | 46 | 11.4 | 17 | 4.4 |
