# Supplementary material for: Collectivism and meaning-making: A search for moderators
Source: PLoS One. 2026 Apr 30;21(4):e0346979. doi: 10.1371/journal.pone.0346979 (PMC13132207; doi:10.1371/journal.pone.0346979)
Supplement: S3 Table — (DOCX) [file pone.0346979.s003.docx]

|  | Study 2 | | | | Study 3 | | | |
| --- | --- | --- | --- | --- | --- | --- | --- | --- |
|  | Republicans  (*n* = 193) | | Democrats  (*n* = 192) | | Republicans  (*n* = 194) | | Democrats  (*n* = 190) | |
|  | *n* | % | *n* | % | *n* | % | *n* | % |
| *Gender* | | | | | | | | |
| Male | 99 | 51.3 | 70 | 36.5 | 97 | 50.0 | 55 | 28.9 |
| Female | 93 | 48.1 | 113 | 58.9 | 97 | 50.0 | 129 | 67.9 |
| Non-Binary | 1 | 0.5 | 6 | 3.1 | - | - | - | - |
| Transgender | 0 | 0.0 | 2 | 1.0 | - | - | - | - |
| Choose not to answer | 0 | 0.0 | 1 | 0.5 | 0 | 0.0 | 0 | 0.0 |
| Another description | - | - | - | - | 0 | 0.0 | 6 | 3.2 |
| *Race-Ethnicity* | | | | | | | | |
| Non-Hispanic White | 142 | 73.6 | 128 | 66.7 | 150 | 77.3 | 122 | 64.2 |
| Black, African American, Afro-Caribbean | 22 | 11.4 | 16 | 8.3 | 18 | 9.3 | 28 | 14.7 |
| Latino or Hispanic | 15 | 7.8 | 15 | 7.8 | 6 | 3.1 | 10 | 5.3 |
| East Asian | 6 | 3.1 | 11 | 3.1 | 10 | 5.2 | 12 | 6.3 |
| South Asian | 0 | 0.0 | 9 | 4.7 | 2 | 1.0 | 5 | 2.6 |
| Middle Eastern or Arab | 1 | 0.5 | 2 | 1.0 | 1 | 0.5 | 0 | 0.0 |
| Native American or Alaskan Native | 1 | 0.5 | 2 | 1.0 | 5 | 2.6 | 0 | 0.0 |
| Mixed | 5 | 2.6 | 8 | 4.2 | 2 | 1.0 | 10 | 5.3 |
| Other | 1 | 0.5 | 1 | 0.5 | 0 | 0.0 | 3 | 1.6 |
