# Supplementary material for: Collectivism and meaning-making: A search for moderators
Source: PLoS One. 2026 Apr 30;21(4):e0346979. doi: 10.1371/journal.pone.0346979 (PMC13132207; doi:10.1371/journal.pone.0346979)
Supplement: S4 Table — Items were 1: In general, I accept the decisions made by my group. 2: When I try to understand an event, the first thing that I consider is its implications for my group -- the people who I care about. 3: It often happens that the interests of my group coincide with my own interests. 4: Whatever is good for my group is good for me. 5: If you know what groups I belong to, you know who I am. 6: I tried to understand the needs and wants of my group and act to fulfill them. (DOCX) [file pone.0346979.s004.docx]

| Scale Item Number | Item Means and *SD*s | | Item Correlations | | | | | |
| --- | --- | --- | --- | --- | --- | --- | --- | --- |
|  | *M* | *SD* | 1 | 2 | 3 | 4 | 5 | 6 |
| Study 1 | | | | | | | | |
| 1 | 4.56 | 1.23 | 1 |  |  |  |  |  |
| 2 | 4.90 | 1.19 | .35 | 1 |  |  |  |  |
| 3 | 4.88 | 1.20 | .25 | .25 | 1 |  |  |  |
| 4 | 3.81 | 1.40 | .45 | .36 | .26 | 1 |  |  |
| 5 | 4.14 | 1.39 | .36 | .35 | .28 | .39 | 1 |  |
| 6 | 5.15 | 1.10 | .35 | .42 | .36 | .35 | .29 | 1 |
| Study 2 | | | | | | | | |
| 1 | 4.80 | 1.13 | 1 |  |  |  |  |  |
| 2 | 4.86 | 1.36 | .34 | 1 |  |  | = |  |
| 3 | 5.11 | 1.18 | .33 | .24 | 1 |  |  |  |
| 4 | 4.38 | 1.35 | .51 | .37 | .27 | 1 |  |  |
| 5 | 4.28 | 1.56 | .47 | .36 | .36 | .55 | 1 |  |
| 6 | 5.15 | 1.20 | .44 | .47 | .36 | .39 | .42 | 1 |
| Study 3 | | | | | | | | |
| 1 | 4.72 | 1.21 | 1 |  |  |  |  |  |
| 2 | 4.82 | 1.39 | .47 | 1 |  |  |  |  |
| 3 | 5.18 | 1.20 | .35 | .31 | 1 |  |  |  |
| 4 | 4.42 | 1.40 | .56 | .39 | .36 | 1 |  |  |
| 5 | 4.48 | 1.53 | .45 | .43 | .38 | .50 | 1 |  |
| 6 | 5.08 | 1.19 | .49 | .55 | .36 | .47 | .45 | 1 |
| Pooled Data | | | | | | | | |
| 1 | 4.69 | 1.20 | 1 |  |  |  |  |  |
| 2 | 4.86 | 1.31 | .39 | 1 |  |  |  |  |
| 3 | 5.06 | 1.20 | .31 | .26 | 1 |  |  |  |
| 4 | 4.20 | 1.41 | .51 | .36 | .31 | 1 |  |  |
| 5 | 4.30 | 1.50 | .42 | .38 | .35 | .49 | 1 |  |
| 6 | 5.13 | 1.16 | .42 | .48 | .36 | .39 | .38 | 1 |
