# Supplementary material for: Collectivism and meaning-making: A search for moderators
Source: PLoS One. 2026 Apr 30;21(4):e0346979. doi: 10.1371/journal.pone.0346979 (PMC13132207; doi:10.1371/journal.pone.0346979)
Supplement: S5 Table — (DOCX) [file pone.0346979.s005.docx]

| Model | *N* (obs/grps) | *R²* Marginal | *R²* Conditional | ICC | AIC | BIC |
| --- | --- | --- | --- | --- | --- | --- |
| Study 1 Students | 808/404 | .053 | .759 | .746 | 2223.1 | 2251.3 |
| Study 2 |  |  |  |  |  |  |
| Full Sample | 770/385 | .052 | .790 | .778 | 2266.7 | 2294.5 |
| Democrats Only | 384/192 | .029 | .840 | .835 | 1052.7 | 1076.4 |
| Republicans Only | 386/193 | .073 | .734 | .713 | 1197.4 | 1221.2 |
| Study 3 |  |  |  |  |  |  |
| Full Sample | 384/768 | .033 | .807 | .800 | 2209.4 | 2237.3 |
| Democrats Only | 190/380 | .050 | .746 | .733 | 1049.1 | 1072.7 |
| Republicans Only | 184/388 | .027 | .845 | .840 | 1151.6 | 1175.4 |
