# Supplementary material for: Collectivism and meaning-making: A search for moderators
Source: PLoS One. 2026 Apr 30;21(4):e0346979. doi: 10.1371/journal.pone.0346979 (PMC13132207; doi:10.1371/journal.pone.0346979)
Supplement: S6 Table — (DOCX) [file pone.0346979.s006.docx]

| Study | In-group | | Out-group | | Statistics | | |
| --- | --- | --- | --- | --- | --- | --- | --- |
|  | *M* | *SD* | *M* | *SD* | *df* | *t* | *p* |
| Study 1 Students | 3.86 | 1.18 | 3.47 | 1.15 | 805.5 | 4.79 | < .001 |
| Study 2 Republicans | 3.56 | 1.40 | 3.18 | 1.32 | 382.8 | 2.73 | < .001 |
| Study 2 Democrats | 3.03 | 1.29 | 2.70 | 1.22 | 380.6 | 2.57 | < .001 |
| Study 3 Republicans | 3.67 | 1.41 | 3.54 | 1.44 | 385.78 | 0.90 | .371 |
| Study 3 Democrats | 3.35 | 1.18 | 3.02 | 1.11 | 376.57 | 2.83 | .005 |
