# Supplementary material for: Collectivism and meaning-making: A search for moderators
Source: PLoS One. 2026 Apr 30;21(4):e0346979. doi: 10.1371/journal.pone.0346979 (PMC13132207; doi:10.1371/journal.pone.0346979)
Supplement: S7 Table — (DOCX) [file pone.0346979.s007.docx]

| Study | In-group | | Out-group | | Statistics | | | |
| --- | --- | --- | --- | --- | --- | --- | --- | --- |
|  | *M* | *SD* | *M* | *SD* | *df* | *t* | *p* | BF₁₀ |
| Study 1 Students | .52 | .27 | .44 | .26 | 804.3 | 3.95 | < .001 | - |
| Study 2 Republicans | .34 | .26 | .34 | .26 | 383.8 | 0.07 | .948 | 0.11 |
| Study 2 Democrats | .42 | .25 | .41 | .27 | 380.3 | 0.28 | .777 | 0.12 |
| Study 3 Republicans | .34 | .28 | .34 | .27 | 385.0 | 0.12 | .901 | 0.08 |
| Study 3 Democrats | .45 | .25 | .44 | .24 | 377.6 | 0.61 | .545 | 0.24 |
