# Supplementary material for: Collectivism and meaning-making: A search for moderators
Source: PLoS One. 2026 Apr 30;21(4):e0346979. doi: 10.1371/journal.pone.0346979 (PMC13132207; doi:10.1371/journal.pone.0346979)
Supplement: S8 Table — (DOCX) [file pone.0346979.s008.docx]

| Study | In-group | | Out-group | | Statistics | | | |
| --- | --- | --- | --- | --- | --- | --- | --- | --- |
|  | *M* | *SD* | *M* | *SD* | *df* | *t* | *p* | BF₁₀ |
| Study 1 Students | .32 | .24 | .23 | .21 | 793.6 | 5.67 | < .001 | - |
| Study 2 Republicans | .32 | .24 | .30 | .22 | 382.3 | 0.71 | .478 | 0.14 |
| Study 2 Democrats | .27 | .25 | .29 | .24 | 382.0 | -0.97 | .332 | 0.18 |
| Study 3 Republicans | .34 | .23 | .32 | .24 | 385.4 | 0.65 | .513 | 0.14 |
| Study 3 Democrats | .29 | .22 | .33 | .23 | 376.87 | -1.59 | .112 | 0.39 |
