# Supplementary material for: Collectivism and meaning-making: A search for moderators
Source: PLoS One. 2026 Apr 30;21(4):e0346979. doi: 10.1371/journal.pone.0346979 (PMC13132207; doi:10.1371/journal.pone.0346979)
Supplement: S9 Table — ΔR² = change model adding study controls vs. models without control; all models include study as a control variable with Study 1 as the reference category. R²adj not reported for RQ2 mixed-effects model due to the complexity of variance partitioning with random effects. Group: Ingroup = 0, Outgroup = 1. Condition: Accuracy = 0, Meaning = 1. RQ2 Mixed-effects model accounts for repeated measures within participants. (DOCX) [file pone.0346979.s009.docx]

| Model | Predictor | *b* | *t* | *p* | *R^2^* adj | *ΔR^2^* |
| --- | --- | --- | --- | --- | --- | --- |
| RQ1: Collectivism to Meaning-Making | | | | | | |
| Linear Regression |  |  |  |  | .034 | .012 |
|  | Collectivism | 0.24 | 6.27 | < .001 |  |  |
|  | Study 2 | -0.59 | -7.04 | < .001 |  |  |
|  | Study 3 | -0.32 | -3.75 | < .001 |  |  |
| RQ2: Group Moderation | | | | | | |
| Mixed-Effects |  |  |  |  | - | .035 |
|  | Collectivism | 0.27 | 6.64 | < .001 |  |  |
|  | Group (Outgroup) | -0.33 | -13.33 | < .001 |  |  |
|  | Collectivism x Group | -0.09 | -3.17 | .002 |  |  |
|  | Study 2 | -0.59 | -7.05 | < .001 |  |  |
|  | Study 3 | -0.32 | -3.75 | < .001 |  |  |
| Simple Slopes |  |  |  |  |  |  |
|  | Ingroup | 0.28 | 6.97 | < .001 |  |  |
|  | Outgroup | 0.20 | 4.85 | < .001 |  |  |
| RQ3: Seeking Meaning Condition | | | | | | |
| Linear Regression |  |  |  |  | .064 | .024 |
|  | Collectivism | 0.27 | 5.27 | < .001 |  |  |
|  | Condition (Meaning) | 0.10 | 1.49 | .164 |  |  |
|  | Collectivism x Condition | -0.07 | -0.95 | .342 |  |  |
|  | Study 2 | -0.60 | -7.06 | < .001 |  |  |
|  | Study 3 | -0.31 | -3.72 | < .001 |  |  |
| RQ4: Content Recall | | | | | | |
| Linear Regression |  |  |  |  | .066 | .043 |
|  | Collectivism | 0.30 | 4.42 | < .001 |  |  |
|  | Content Recall | -0.26 | -1.92 | .055 |  |  |
|  | Collectivism x Content | -0.18 | -1.20 | .232 |  |  |
|  | Study 2 | -0.63 | -7.34 | < .001 |  |  |
|  | Study 3 | -0.35 | -4.07 | < .001 |  |  |
| RQ4: Source Recall | | | | | | |
| Linear Regression |  |  |  |  | .090 | .067 |
|  | Collectivism | 0.12 | 1.95 | .052 |  |  |
|  | Source Recall | 0.96 | 5.84 | < .001 |  |  |
|  | Collectivism x Source | 0.29 | 1.72 | .086 |  |  |
|  | Study 2 | -0.61 | -7.34 | < .001 |  |  |
|  | Study 3 | -0.36 | -4.28 | < .001 |  |  |
