# Supplementary material for: Collectivism and meaning-making: A search for moderators
Source: PLoS One. 2026 Apr 30;21(4):e0346979. doi: 10.1371/journal.pone.0346979 (PMC13132207; doi:10.1371/journal.pone.0346979)
Supplement: S10 Table — Note. ICC = the study-level variance proportion after accounting for participant clustering All models included random intercepts for Study. For RQ2, the model also included random intercepts for participants. (DOCX) [file pone.0346979.s010.docx]

| Research Questions | Predictor | *b* | *t* | *p* | ICC |
| --- | --- | --- | --- | --- | --- |
| RQ1: Collectivism to Meaning-Making |  |  |  |  | .051 |
|  | Collectivism | 0.24 | 6.24 | <.001 |  |
| RQ2: Group Moderation |  |  |  |  | .051 |
|  | Collectivism | 0.28 | 6.94 | <.001 |  |
|  | Group (Outgroup) | -0.33 | -13.32 | <.001 |  |
|  | Collectivism x Group | -0.09 | -3.17 | .002 |  |
| RQ3: Seeking Meaning Condition |  |  |  |  | .058 |
|  | Collectivism | 0.27 | 5.25 | <.001 |  |
|  | Condition (Meaning) | 0.10 | 1.39 | .165 |  |
|  | Collectivism x Condition | -0.72 | -0.95 | .344 |  |
| RQ4: Content Recall |  |  |  |  | .062 |
|  | Collectivism | 0.30 | 4.39 | <.001 |  |
|  | Content Recall | -0.26 | 1.88 | .060 |  |
|  | Collectivism x Content | -0.17 | -1.18 | .240 |  |
| RQ4: Source Recall | | | | | .059 |
|  | Collectivism | 0.12 | 1.94 | .052 |  |
|  | Source Recall | 0.96 | 5.84 | <.001 |  |
|  | Collectivism x Source | 0.29 | 1.71 | .087 |  |
