# Supplementary material for: Collectivism and meaning-making: A search for moderators
Source: PLoS One. 2026 Apr 30;21(4):e0346979. doi: 10.1371/journal.pone.0346979 (PMC13132207; doi:10.1371/journal.pone.0346979)
Supplement: S11 Table — (DOCX) [file pone.0346979.s011.docx]

| Model | Predictor | *b* | 95% CI | *p* |
| --- | --- | --- | --- | --- |
| RQ1: Collectivism to Meaning-Making | | | | |
|  | Full Pooled | 0.24 | [.16, .31] | < .001 |
|  | Drop Study 1 | 0.25 | [.15, .34] | < .001 |
|  | Drop Study 2 | 0.23 | [.14, .31] | < .001 |
|  | Drop Study 3 | 0.25 | [.15, .34] | < .001 |
| RQ2: Group Moderation: Collectivism x Group | | | | |
|  | Full Pooled | -0.09 | [-.14, -.03] | .002 |
|  | Drop Study 1 | -0.09 | [-.16, -.03] | .005 |
|  | Drop Study 2 | -0.06 | [-.12, .00] | .058 |
|  | Drop Study 3 | -0.11 | [-.18, -.04] | .001 |
| RQ3: Seeking Meaning Condition: Collectivism x Condition | | | | |
|  | Full Pooled | -0.08 | [-.22, .08] | .323 |
|  | Drop Study 1 | -0.06 | [-.24, .13] | .553 |
|  | Drop Study 2 | -0.12 | [-.30, .06] | .176 |
|  | Drop Study 3 | -0.05 | [-.23, .14] | .612 |
| RQ4:Collectivism x Content Recall | | | | |
|  | Full Pooled | -0.18 | [-.47, .11] | .202 |
|  | Drop Study 1 | -0.28 | [-.66, .10] | .144 |
|  | Drop Study 2 | -0.01 | [-.36, .33] | .940 |
|  | Drop Study 3 | -0.28 | [-.62, .06] | .109 |
| RQ4: Collectivism x Source Recall | | | | |
|  | Full Pooled | 0.29 | [-.04, .62] | .086 |
|  | Drop Study 1 | 0.27 | [-.14, .68] | .197 |
|  | Drop Study 2 | 0.31 | [-.08, .69] | .121 |
|  | Drop Study 3 | 0.26 | [-.16, .68] | .221 |
