# Supplementary material for: Collectivism and meaning-making: A search for moderators
Source: PLoS One. 2026 Apr 30;21(4):e0346979. doi: 10.1371/journal.pone.0346979 (PMC13132207; doi:10.1371/journal.pone.0346979)
Supplement: S12 Table — In linear regression models, power was based on increase in explained variance (ΔR²) when adding the predictor to the model expressed as Cohen’s f². RQ2 power for the mixed-model interaction estimated using simr::powerSim (n = 1,000). (DOCX) [file pone.0346979.s012.docx]

| Model | Power | | | |
| --- | --- | --- | --- | --- |
|  | Pooled Data | Study 1 | Study 2 | Study 3 |
| RQ1: Collectivism to Meaning-Making | 1.00 | .93 | .97 | .94 |
| RQ2: Collectivism x Group | .88 | .49 | .77 | .24 |
| RQ3: Collectivism x Condition | .16 | .23 | .06 | .17 |
| RQ4:Collectivism x Content Recall | .22 | .10 | .50 | .05 |
| RQ4: Collectivism x Source Recall | .41 | .11 | .09 | .23 |
