# Supplementary material for: Collectivism and meaning-making: A search for moderators
Source: PLoS One. 2026 Apr 30;21(4):e0346979. doi: 10.1371/journal.pone.0346979 (PMC13132207; doi:10.1371/journal.pone.0346979)
Supplement: S1 File — (DOCX) [file pone.0346979.s013.docx]

**Preliminary Analyses: In-Group/Out-Group Validation**

**Pilot.** For University of Southern California (USC) undergraduate students, identification score for a Trojan was above the neutral midpoint “4” on a 7-point scale (*M* = 6.35, *SD* = 0.97), *t*(204) = 34.82, *p* < .001, and identification score for a Bruin was significantly lower than the neutral midpoint (*M* = 1.15, *SD* = 0.64), *t*(204) = -63.82, *p* < .001, with a significant difference between the two scores, *t*(354.03) = 64.27, *p* < .001.

**Study 1.** For USC undergraduates, identification score for a Trojan was above the neutral midpoint “4” on a 7-point scale (*M* = 6.18, *SD* = 1.03), *t*(404) = 42.60, *p* < .001, and identification score for a Bruin was significantly lower than the neutral midpoint (*M* = 1.12, *SD* = 0.54), *t*(505) = -107.19, *p* < .001, with a significant difference between the two scores, *t*(611.37) = 87.58, *p* < .001.

**Study 2.** For Republicans, identification score for a Trump Supporter was above the neutral midpoint “4” on a 7-point scale (*M* = 5.39, *SD* = 1.73), *t*(192) = 11.18, *p* < .001, and identification score for a Harris Supporter was significantly lower than the neutral midpoint (*M* = 1.53, *SD* = 1.20), *t*(192) = -38.47, *p* < .001, with a significant difference between the two scores, *t*(343) = 25.34, *p* < .001. For Democrats, identification score for a Harris Supporter was above the neutral midpoint (*M* = 5.49, *SD* = 1.76), *t*(191) = 11.75, *p* < .001, and identification score for a Trump Supporter was significantly lower than the neutral midpoint (*M* = 1.27, *SD* = 0.96), *t*(191) = -39.54, *p* < .001, with a significant difference between the two scores, *t*(294.78) = 29.20, *p* < .001.

**Study 3.** For Republicans, identification score for a Trump Supporter was above the neutral midpoint “4” on a 7-point scale (*M* = 5.28, *SD* = 1.76), *t*(193) = 10.18, *p* < .001, and identification score for a Democrat was significantly lower than the neutral midpoint (*M* = 1.67, *SD* = 1.28), *t*(193) = -25.33, *p* < .001, with a significant difference between the two scores, *t*(353.09) = 23.15, *p* < .001. For Democrats, identification score for a Democrat was above the neutral midpoint (*M*=5.24, *SD*=1.71), *t*(189)=9.99, *p* < .001, and identification score for a Trump Supporter was significantly lower than the neutral midpoint (*M*=1.18, *SD*=0.59), *t*(189) = -65.41, *p* < .001, with a significant difference between the two scores, *t*(233.7)=30.85, *p* < .001.
