# Supplementary material for: Collectivism and meaning-making: A search for moderators
Source: PLoS One. 2026 Apr 30;21(4):e0346979. doi: 10.1371/journal.pone.0346979 (PMC13132207; doi:10.1371/journal.pone.0346979)
Supplement: S2 File — (DOCX) [file pone.0346979.s014.docx]

**Study 1 Replication**

We pre-registered our *Study 1-Replication* analyses at <https://aspredicted.org/hsc8-nyfy.pdf>.

**Participants**

Undergraduates participated for university subject pool credit (*N* = 396, *n* = 386; 64.2% female; Age *M* = 20.19, *SD* = 1.86; see Table S2).

**Results and Discussion**

**Preliminary Analyses:** USC undergrads scored above the neutral midpoint “4” on a 7-point scale (*M*=6.23, *SD*=1.03), *t*(385)=42.32, *p* < .001, identifying as a Trojan (USC mascot), and below the scale neutral midpoint (*M*=2.15, *SD*=1.09), *t*(385)=-144.83, *p* < .001) as a Bruin (UCLA mascot), with a significant difference in university identification, *t*(495.14) = 91.24, *p* < .001.

**RQ1:** Supporting RQ1, collectivism score positively correlated with meaningfulness ratings, *r*(384) = .17, *p* = .001.

**RQ2:** To test whether group membership moderates the relationship between collectivism and meaning-making, we conducted a mixed-effects regression analysis, accounting for repeated measurements. We found significant main effects (collectivism *b* = 0.20, *p* = .002; communicator group membership *b* = -0.09, *p* < .001, collectivism x group membership *b* = -0.03, *p* = .187), suggesting collectivism predicted meaning-making regardless of message source, as observed in Study 1.

**RQ3:** To test whether random assignment to instruction to seek meaning (accuracy) moderates the relationship between collectivism and meaning-making, we conducted a regression analysis including collectivism (*b* = 0.12, *p* = .164), condition (*b* = 0.00, *p* = .990), and their interaction (*b* = 0.16, *p* = .174) as predictors. Neither main nor interaction effects were statistically significant; a follow-up Bayesian analysis yielded BF₁₀ = 0.40, providing moderate evidence for the null hypothesis that random assignment did not affect the relationship between collectivism and meaning-making.

**RQ4**: To examine whether depth of processing moderates the relationship between collectivism and meaning-making, we conducted two separate moderation analyses focusing on content recall (corrected hit rates) and source recall. The content recall regression analysis revealed main effects of collectivism (*b* = 0.18, *p* = .021) and content recall (*b* = -0.49, *p* < .001, collectivism x content recall, *b* = -0.02, *p* = .885); a follow-up Bayesian analysis yielded BF₁₀ = 0.14, providing substantial evidence for the null hypothesis that content recall does not moderate the collectivism-meaning relationship. The source recall regression analysis revealed a main and interaction effect of collectivism (collectivism *b* = 0.31, *p* < .001, source recall accuracy *b* = 0.28, *p* = .127, collectivism x source recall *b* = -0.46, *p* = .039). Simple slopes analysis revealed that collectivism strongly predicted meaning-making when source recall was low (*b* = 0.31, *p* < .001), not high (*b* = -0.15, *p* = .360), implying that collectivistic tendencies influence meaning-making primarily when source information is processed less thoroughly.
